# Use of withdrawal management services among people who use illicit drugs in Vancouver, Canada

**DOI:** 10.1186/s13011-018-0164-3

**Published:** 2018-07-05

**Authors:** Sharon Vipler, Kanna Hayashi, M.-J. Milloy, Evan Wood, Ekaterina Nosova, Thomas Kerr, Lianping Ti

**Affiliations:** 1 British Columbia Centre for Excellence in HIV/AIDS, University of British Columbia, St. Paul’s Hospital, 608-1081 Burrard Street, Vancouver, BC V6Z 1Y6 Canada; 2 BC Centre on Substance Use, 400-1045 Howe Street, Vancouver, BC V6Z 2A9 Canada; 3 Faculty of Health Sciences, Simon Fraser University, 8888 University Drive, Burnaby, BC V5A 1S6 Canada; 4 Department of Medicine, University of British Columbia, St. Paul’s Hospital, 608-1081 Burrard Street, Vancouver, BC V6Z 1Y6 Canada; 5 Department of Family Practice, University of British Columbia, 320 – 5950 University Boulevard, Vancouver, BC V6T 1Z6 Canada

**Keywords:** Detoxification services, Withdrawal management, Addiction treatment, People who use illicit drugs

## Abstract

**Background:**

For many individuals with substance use disorders, the entry point for addiction treatment can be through withdrawal management (e.g. detoxification) services. However, little is known about the factors that predict withdrawal management service use among people who use illicit drugs (PWUD). Using data derived from two prospective cohorts of PWUD, we conducted a longitudinal data analysis of factors associated with use of withdrawal management services.

**Methods:**

Individuals participating in two cohorts of PWUD were prospectively followed between December 2005 and May 2016 in Vancouver, Canada. Bivariate and multivariate generalized estimating equations were used to examine factors associated with use of withdrawal management services.

**Results:**

Out of a total of 2001 participants, 339 (16.9%) individuals reported having been to a withdrawal management centre in the previous 6 months at some point during the study period. In multivariate analyses, male sex (adjusted odds ratio [AOR]: 1.62, 95% Confidence Interval [CI]: 1.17–2.24), homelessness (AOR: 1.86, 95% CI: 1.45–2.38), binge use of any substance (AOR: 1.34, 95% CI: 1.08–1.67), having attended a supervised injection facility (AOR: 1.66, 95% CI: 1.3–2.11), and having accessed other addiction medicine treatment or supports (other than withdrawal management services or opioid agonist therapy; AOR: 3.34, 95% CI: 2.64–4.22) were positively associated with having accessed withdrawal management services, whereas older age (AOR: 0.81, 95% CI: 0.7–0.94) was negatively associated with the outcome.

**Conclusions:**

This study identified specific factors associated with accessing withdrawal management services. Current evidence suggests a need to re-examine the provision of withdrawal management services. Consideration needs to be given to redesigning access to care and bridging to evidence-based addiction treatment, particularly for highly vulnerable subpopulations, identified in this study as females and older people.

## Background

People who use illicit drugs (PWUD) are vulnerable to an array of health-related harms. For example, the use of opioids carries high risk for overdose and death and currently in many North American jurisdictions, the rate of opioid associated overdose deaths is being regarded as public health crises [1–3]. Misuse of stimulants have been associated with seizures, hemorrhagic and ischemic strokes, cardiac arrhythmias, myocardial infarctions, ischemic colitis and intestinal ischemia [4, 5]. Moreover, alcohol is not without harmful effects and use is highly prevalent among PWUD [6, 7]. Use has been linked to liver cirrhosis, cardiovascular disease, injuries due to violence or motor vehicle collisions, fetal alcohol syndrome and pre-term birth complications [8–10].

Treatment for substance use disorders range from medical to psychosocial, flexible to structured and from outpatient options to intensive inpatient programs [11]. Withdrawal management facilities (previously more commonly referred to as detoxification centres) can be the first point of contact for individuals seeking assistance in the management of their substance use disorders. For many substance use disorders, acute withdrawal management cannot be considered a stand-alone treatment [12–14]. However, for many individuals, it is a vital first step in connecting patients to more definitive treatment options, including but not restricted to opioid agonist therapy and alcohol relapse prevention medications [11]. In general, withdrawal management facilities provide assistance in the mitigation of withdrawal symptoms through medical supervision and access to pharmacological treatment options [11]. In some cases, medical withdrawal management facilities can initiate definitive management in the form of agonist therapy (opioid use disorder) or relapse prevention medications (alcohol use disorders) [15]. For example, in British Columbia, Canada, access to withdrawal management facilities is predominantly through a process of self-referral [16–18]. On demand access to withdrawal management is not yet available. Most individuals are placed on a waitlist following self-referral. Occasionally, individuals will be transferred from acute care hospitals to withdrawal management facilities.

Studies consistently demonstrate that only 10–15% of individuals diagnosed with substance use disorders access addiction treatment in a given year [19]. Literature on access to addiction treatment has largely explored barriers to accessing services, which includes those individuals with unstable housing, individuals engaged in sex work and individuals with concurrent mental health diagnoses [19–22]. However, these studies focused broadly on addiction treatment in general, and little is known about the characteristics of those who access withdrawal management services specifically. The present study sought to examine which factors were associated with attendance at withdrawal management services among a cohort of socially disadvantaged PWUD in in urban open drug scene setting in the poorest postal code in the country, in Vancouver, Canada.

## Methods

### Study design

Data for this study were derived from the Vancouver Injection Drug Users Study (VIDUS) and AIDS Care Cohort to evaluate Exposure to Survival Services (ACCESS) cohorts. These studies are open prospective cohorts of PWUD in Vancouver. VIDUS started in 1996 and includes participants who were 18 years old or older, lived in the greater Vancouver region and had used injection drugs in the 1 month prior to baseline interview. In 2005, the participants were divided into two studies with the HIV-positive participants being followed by ACCESS and the HIV-negative participants being followed by VIDUS. Additionally, the ACCESS cohort was expanded to include people who use illicit drugs other than or in addition to cannabis in the month prior to baseline interview. Recruitment and follow-up procedures were harmonized between VIDUS and ACCESS cohorts to facilitate analyses of merged data. Detailed descriptions of the cohorts are described elsewhere [23, 24]. Primary methods of recruitment were through self-referral, word of mouth, street outreach and snowball sampling. All participants provided written informed consent.

Study participants completed interviewer-administered questionnaires at enrollment and every 6 months thereafter. The questionnaire covered various topics, such as demographic information, injection/non-injection drug use, access to addiction treatment services and risk behaviours. At each visit, blood samples were drawn for HIV and Hepatitis C testing, as well as HIV disease monitoring for ACCESS participants. Interviews were conducted in private and participants had access to pretest and post-test counseling with nurses. Participants were provided with a $30 (Canadian) stipend each visit to cover their time and transportation costs. Ethics approval for VIDUS and ACCESS was provided by the University of British Columbia/Providence Health Care Research Ethics Board. The study sample was restricted to all participants who completed a baseline survey between December 2005 and May 2016.

### Variable selection

In the present study, the outcome of interest was whether a participant had accessed withdrawal management services in the previous 6 months. Accessing withdrawal management services was defined as having initiated but not necessarily completed a course of withdrawal management. Explanatory variables were selected based on the previous literature indicating factors associated with accessing addiction treatment in general [19–22]. These included: sex (male vs. female); white ethnicity/ancestry; age (per 10 year increase); homelessness; in a stable relationship (defined as same partner greater than 3 months); incarceration; regular employment; sex work; daily heroin use; daily cocaine use; daily crystal methamphetamine use; daily prescription opioid use; high-risk alcohol use (defined as per the National Institute on Alcohol Abuse and Alcoholism as more than three drinks per day or seven drinks per week for females and more than four drinks per day or 14 drinks per week for males) [25]; binge drug use, defined as having used illicit drugs more than usual; injection use of drugs; overdose; having accessed a supervised injection facility; admission to hospital; having a psychiatric illness; having been on opioid agonist therapy, defined as on a buprenorphine/naloxone maintenance program or methadone maintenance treatment (MMT) program; and having been in any other form of treatment and supports (excluding withdrawal management services or opioid agonist therapy). All behavioural variables referred to the six-month period prior to the follow-up interview and were treated as time-varying variables unless otherwise indicated.

### Statistical analysis

As a first step, we used Pearson’s Chi Square test and Mann-Whitney test to examine the baseline sample characteristics strbatified by having accessed withdrawal management services in the previous 6 months at baseline. To identify factors independently associated with accessing withdrawal management services in the previous 6 months, a multivariate generalized estimating equation (GEE) model was created based on examination of the quasi-likelihood under the independence model criterion (QIC) for GEE and *p*-values. First, we constructed a full model that included all variables significant at *p* < 0.10 in bivariate GEE analyses. After noting the QIC of the model, we removed the variable with the largest p-value and built a reduced model. This iterative process continued until no variables remained and we selected the multivariate model with the lowest QIC score. Given the large number of candidate explantory variables and to address the potential for Type 1 error, we used an adjusted significance level alpha of 0.01. All significant *p*-values were < **0.01.** We also quantified the severity of multicollinearity using the variance inflation factor (VIF). All p-values were two sided. All statistical analyses were conducted using R version 0.99.892 (R Foundation for Statistical Computing, Vienna, Austria). We restricted the sample to observations when the status of access to withdrawal management was known and then ran bivariate models. The maximum percentage of missing observations for each variable included in the GEE was less than 1%. The total number of observations containing a missing value in at least one of the variables included in the GEE was 431 (2.1%).

## Results

In total, 2001 participants from VIDUS and ACCESS were included, of which 685 (34.2%) were female and 1179 (58.9%) were white. The median age at enrollment was 41 years (quartile [Q]1 – Q3: 34.6–47.6 years). Median follow-up length of the sample was 78.8 months (Q1 – Q3: 26.5–114.2 months), for a total of 11,809 person-years of follow-up. In total, 69 participants reported having accessed withdrawal management services at baseline and 339 participants (16.9%) reported having accessed withdrawal management services at least once during the study. For the 339 individuals, the median number of times withdrawal management services were accessed was 1, with an IQR range of 1–2. At baseline, 27% of respondents were daily heroin users and 6.2% were daily prescription opioid users. 9.2% were daily cocaine users and 6% were daily crystal methamphetamine users. 12.5% of participants were engaging in high risk alcohol use and 40% were noted to engage in binge use (of any substance). 89.7% of the participants were injection drug users. Table 1 details baseline characteristics of participants, stratified by those who did and did not access withdrawal management services in the previous 6 months.

**Table 1:** Baseline characteristics of people who use drugs enrolled in the VIDUS and ACCESS cohorts, stratified by use of withdrawal management services in the previous 6 months, Vancouver, Canada, Dec 2005 to May 2016

Characteristic	Total (%) (*n* = 2001)	Use of Withdrawal Management Services		*p* – value**
		Yes (*%*) (*n* = 69)	No (%) (*n* = 1932)	
Male	1313 (65.6)	49 (71.0)	1264 (65.4)	0.262
White ethnicity	1179 (58.9)	45 (65.2)	1134 (58.7)	0.279
Age (median, Q1-Q3)	41.5 (34.6–47.6)	39.8 (32.0–45.6)	41.6 (34.9–47.7)	0.037
Homelessness*	718 (35.9)	35 (50.7)	638 (35.4)	0.009
In a stable relationship	578 (28.9)	19 (27.5)	559 (28.9)	0.754
Incarceration*	331 (16.5)	12 (17.4)	319 (16.5)	0.854
Regular Employment*	474 (23.7)	22 (31.9)	452 (23.4)	0.103
Sex Work*	315 (15.7)	12 (17.4)	303 (15.7)	0.713
Daily Heroin Use*	540 (27.0)	25 (36.2)	515 (26.7)	0.080
Daily Cocaine Use*	184 (9.2)	11 (15.9)	173 (9.0)	0.049
Daily Methamphetamine Use*	121 (6.0)	9 (13.0)	112 (5.8)	0.013
Daily Prescription Opioid Use*	125 (6.2)	5 (7.2)	120 (6.2)	0.728
High Risk Alcohol Use*†	251 (12.5)	6 (8.7)	245 (12.7)	0.325
Binge Use (any substance)*	801 (40.0)	34 (49.3)	767 (39.7)	0.118
Injection Drug Use (any substance)*	1795 (89.7)	64 (92.8)	1731 (89.6)	0.402
Overdose*	152 (7.6)	11 (15.9)	141 (7.3)	0.008
Use at Supervised Injection Facility*	991 (49.5)	41 (59.4)	950 (49.2)	0.103
Hospital Admission*	422 (21.1)	18 (26.1)	404 (20.9)	0.300
Mental Health diagnosis	988 (49.4)	44 (63.8)	944 (48.9)	0.015
Opioid Agonist Therapy*	837 (41.8)	20 (29.0)	817 (42.3)	0.027
Other Addiction Treatment/Supports*‡	298 (14.9)	37 (53.6)	261 (13.5)	< 0.001

* in the last 6 months

† as per NIAAA guidelines (> 3 drinks/day or 7 drinks/week for females and > 4 drinks/day or 14 drinks/week for males)

‡ other than withdrawal management services or opioid agonist therapy

** Pearson’s Chi Square test and Mann-Whitney test were used to examine the baseline sample characteristics

Bivariate GEE analyses indicated factors associated with accessing withdrawal management services among PWUD (Table 2). Injection drug use and attendance at a supervised injection facility were found to be collinear and therefore the injection drug use variable was removed prior to conducting multivariate analyses**.** Given that SIFs are not available in many settings, we chose to include the injection drug use variable so that the findings would be more relevant to other settings.

**Table 2:** Bivariate and multivariate GEE analysis of factors associated with use of withdrawal management services in people who use drugs in Vancouver, Canada (n = 2001)

	Unadjusted		Adjusted	
Characteristic	Odds Ratio (95% CI)	*p –* value**	Odds Ratio (95% CI)	*p –* value**
Male				
(yes vs. no)	1.26 (0.97–1.64)	0.084	1.62 (1.17–2.24)	0.004
White Ethnicity				
(yes vs. no)	1.07 (0.84–1.36)	0.599	–	–
Age				
(per 10 year increase)	0.66 (0.59–0.74)	< 0.001	0.81 (0.70–0.94)	0.005
Homelessness*				
(yes vs. no)	2.47 (2.01–3.04)	< 0.001	1.86 (1.45–2.38)	< 0.001
In a stable relationship				
(yes vs. no)	0.82 (0.67–1.01)	0.064	0.91 (0.72–1.14)	0.402
Incarceration*				
(yes vs. no)	1.54 (1.16–2.05)	0.003	–	–
Regular Employment*				
(yes vs. no)	0.98 (0.78–1.24)	0.878	–	–
Sex Work*				
(yes vs. no)	1.58 (1.18–2.12)	0.002	1.42 (0.95–2.11)	0.085
Daily Heroin Use*				
(yes vs. no)	1.58 (1.25–2.01)	< 0.001	–	–
Daily Cocaine Use*				
(yes vs. no)	1.97 (1.45–2.67)	< 0.001	1.40 (0.98–1.99)	0.066
Daily Methamphetamine Use*				
(yes vs. no)	1.33 (0.88–2.01)	0.179	–	–
Daily Prescription Opioid Use*				
(yes vs. no)	1.58 (1.11–2.24)	0.011	1.37 (0.92–2.04)	0.119
High Risk Alcohol Use*^†^				
(yes vs. no)	1.21 (0.94–1.56)	0.143	–	–
Binge Use (any substance)				
(yes vs. no)	1.63 (1.35–1.96)	< 0.001	1.34 (1.08–1.67)	0.009
Injection Drug Use (any substance)*				
(yes vs. no)	2.19 (1.68–2.85)	< 0.001	–	–
Overdose*				
(yes vs. no)	1.70 (1.19–2.44)	0.004	–	–
Use at Supervised Injection Facility*				
(yes vs. no)	2.24 (1.81–2.77)	< 0.001	1.66 (1.30–2.11)	< 0.001
Hospital Admission*				
(yes vs. no)	1.40 (1.13–1.73)	0.002	1.29 (0.99–1.67)	0.061
Mental Health illness				
(yes vs. no)	1.23 (0.96–1.57)	0.101	–	–
Opioid Agonist Therapy				
(yes vs. no)	0.94 (0.75–1.17)	0.556	–	–
Other Addiction Treatment/Supports*^‡^				
(yes vs. no)	3.64 (2.94–4.52)	< 0.001	3.34 (2.64–4.22)	< 0.001

^*^ in the last 6 months

^†^ as per NIAAA guidelines (> 3 drinks/day or 7 drinks/week for females and > 4 drinks/day or 14 drinks/week for males)

^‡^ Other than withdrawal management services or opioid agonist therapy

** A bivariate and multivariate generalized estimating equation (GEE) model was created based on examination of the quasi-likelihood under the independence model criterion (QIC) for GEE and *p*-values

In multivariate analyses (also presented in Table 2), factors that were positively and independently associated with having accessed withdrawal management services included: male sex (AOR: 1.62, 95% CI: 1.17–2.24), homelessness (AOR: 1.86, 95% CI: 1.45–2.38), binge use of any substance (AOR: 1.34, 95% CI: 1.08–1.67), having attended a supervised injection facility (AOR: 1.66, 95% CI: 1.30–2.11), and having accessed other addiction medicine treatment or supports (other than withdrawal management services or opioid agonist therapy; AOR: 3.34, 95% CI: 2.64–4.22). Older age (AOR: 0.81, 95% CI: 0.70–0.94) was negatively and independently associated with having accessed withdrawal management services.

## Discussion

The present study found that only 17% of participants in the study accessed withdrawal management services over a median of 78.8 months of follow-up. The prevalence reported in the present study is consistent with numbers reported using large scale data from the United States [26]. Male sex, homelessness, binge use of any substance, attendance at supervised injection facilities, and having accessed other addiction treatment or supports (other than withdrawal management services or opioid agonist therapy) were positively associated with having accessed withdrawal management services, while older age was found to be negatively associated with having accessed withdrawal management services.

We found that men were 62% more likely to have accessed withdrawal management services, which aligns with published literature that women who use substances have traditionally faced additional barriers to accessing addiction treatment in comparison to men [27–29]. Navigating the intricacies of accessing treatment when a spouse also has a substance use disorder, or when pregnant, or facing the potential for child apprehension has been shown to be powerful deterrents to accessing addiction treatment [28–30]. Of concern, there is evidence to suggest women’s substance use trajectories move faster and that at the time of treatment entry, women have greater medical, psychiatric and adverse social consequences [31].

The finding that homelessness was associated with having accessed withdrawal management services appears to be contrary to the existing literature. In our study, homeless individuals were 86% more likely to have accessed withdrawal management services. Previous findings from Seattle, Washington and Alameda County, California suggest that homeless individuals have greater difficulty accessing treatment for their substance use disorders compared to stably housed individuals [32, 33]. A recent study based on the same study cohort population as the present study found homelessness to be associated with an inability to ever have accessed addiction treatment (implying a desire to access treatment for substance use disorder) [21]. It should be noted that in contrast to the aforementioned studies that examined addiction treatment more generally, our study sought to tease out withdrawal management services as a specific component of addiction treatment. Taken together, the body of literature suggests that while homelessness is associated with barriers to accessing addiction treatment in general, this may not be the case when specifically looking at access to withdrawal management services. It may also be that individuals are using withdrawal management facilities for the purposes of housing respite when lacking access to other options such as shelters [34]. It is not known whether there is a seasonal variation to accessing withdrawal management services. This would be an area for future research, specifically to examine whether there is increased access to withdrawal management facilities in winter and whether that increase was seen specifically in the homeless population. Our setting does not have a pronounced seasonal variation in weather and this should be explored in settings with greater seasonal variation.

Binge use of any substance was associated with 34% increased likelihood of accessing withdrawal management services. Binge use may be linked to a higher severity of addiction, and perhaps a higher need to access addiction treatment. The potential harms associated with binge use of substances may put individuals in more frequent contact with health care providers, increasing the chance of referral. Future research should seek to tease out the relationship between binge use, engagement with healthcare services, and access to withdrawal management services.

A previously well-established link was demonstrated by the present study in that participants who accessed supervised injection sites were 66% more likely to have accessed withdrawal management services. Specifically, an evaluation of a supervised injection site in this setting demonstrated that at least weekly use of the supervised injection site and any contact with counseling services at the site were associated with increased entry into withdrawal management services [35, 36]. Similar results were demonstrated with needle exchange programs connecting individuals with referrals to addiction treatment in Baltimore, USA [33, 37, 38]. A systematic review found that supervised injection services enhanced connections between PWID and addiction treatment and social services [39].

The present study also found that having accessed other addiction treatment programs or supports increased the likelihood of having accessed withdrawal management services by over three times. There is a paucity of literature to address this finding. It may be that certain programs (e.g. treatment facilities) require individuals to attend withdrawal management facilities prior to being granted program entry, as they may not be equipped to manage withdrawal. Potentially, addiction supports such as mutual help groups (e.g., Alcoholics Anonymous) may have networks of individuals able to assist individuals in accessing other resources, including withdrawal management facilities.

Older age was negatively associated with accessing withdrawal management services, with older individuals being 19% less likely to access withdrawal management services. Despite previous theories that individuals “age out” of their substance use disorders, illicit substance use has been found to be a prevailing issue among older adult heroin users [40]. Older homeless adults with substance use disorders have been shown to have higher burden of physical and mental illnesses, which in turn make it harder to meet the daily needs for food, shelter and safety. This daily struggle to address basic survival can shift priorities away from treatment of their substance use [41].

There are a number of public health implications from this study. Our findings indicated a small percentage of people who accessed withdrawal management services, which highlights a need to reexamine the current infrastructure of these services. Evidence has demonstrated the increased risks of mortality of short admissions to withdrawal management facilities as a treatment modality, specifically for individuals with opioid use disorders. [42]. A large body of research recommends a shift towards a system that allows for longer treatment periods, perhaps initiated in a withdrawal management facility and then immediately transitioned to outpatient settings or residential treatment settings [42–45]. As it stands, withdrawal management facilities in many settings are slowly shifting service provision to better reflect the current best evidence. At the time of the present study, most facilities were seeing all substance use disorders with minimal triaging in place. In particular, individuals with opioid use disorder were admitted for short admissions of rapid opioid agonist tapers or short periods of abstinence. As noted above, these approaches increase the risk of morbidity and mortality. For the most part, evidence points to outpatient settings for managing opioid use disorders [46]. Indeed, the current system of care in British Columbia is endeavoring to move towards best practice with the majority of opioid use disorders being managed in an outpatient setting and inpatient withdrawal management facilities being reserved for cases where supervised medical withdrawal is required, such as with alcohol withdrawal. However, exceptions exist (e.g., polysubstance use disorders, treatment facilities requiring formalized withdrawal management prior to admission) and therefore the substance use disorders managed in these facilities will continue to be mixed.

Information regarding the characteristics of those who have accessed withdrawal management services and in what context can be used to better design addiction treatment programs and access to these facilities. For example, waitlists have been shown to decrease the likelihood of accessing addiction treatment [19]. That is an argument for on-demand access to withdrawal management services. Creating an interconnected network of services to allow for seamless transitions between services may improve access and retention in addiction treatment. Patient engagement and further research to best identify the gaps in accessing services, delivery in care and transition to definitive treatment may answer questions left unanswered by this study. This is assuming that accessing withdrawal management impacts the course of substance use disorders. There is a lack of literature to support this assumption, highlighting a potential for future research to examine the effect of accessing withdrawal management services.

There are several limitations to this study. First, we did not randomly sample individuals; therefore, the findings of this study may not be representative of or generalizable to the local community or other populations. Second, the study relied largely on self-report. As such, the findings may be susceptible to recall bias and socially desirable responding though we know of no reason why withdrawal services use would be subjected to socially desirable responding. Lastly, given the observational nature of the study, we could not ascertain causality from the study.

## Conclusion

In summary, our study again highlighted the low prevalence of accessing withdrawal management services. We found that male sex, homelessness, binge use of any substance, attendance at supervised injection facilities and having accessed other addiction treatment or supports (other than withdrawal management services or opioid agonist therapy) were positively associated with having accessed withdrawal management services, while older age was negatively associated with the outcome. Current evidence suggests a need to re-examine the provision of withdrawal management services. Consideration needs to be given to redesigning access to care and bridging to evidence-based addiction treatment, particularly for highly vulnerable subpopulations.
